# Characterization of Secondary Health Conditions Among United States Service Members with Combat-Related Lower Extremity Limb Salvage

**DOI:** 10.3390/jcm14103472

**Published:** 2025-05-15

**Authors:** Sarah R. Franco, Susan L. Eskridge, Stephen M. Goldman, Christopher L. Dearth

**Affiliations:** 1 Extremity Trauma and Amputation Center of Excellence, Defense Health Agency, Falls Church, VA 22042, USA; 2 Department of Surgery, Uniformed Services University of the Health Sciences and Walter Reed National Military Medical Center, Bethesda, MD 20814, USA; 3 Leidos, San Diego, CA 92121, USA

**Keywords:** military medicine, war-related injuries, trauma, extremity injuries

## Abstract

**Background:** Musculoskeletal trauma involving the lower extremities is an unfortunately prevalent injury pattern in contemporary warfare, and the secondary health conditions (SHCs) associated with these injuries remain largely unexplored. **Methods:** U.S. Service members (SMs) with combat-related lower extremity injuries between 2004 and 2014 were categorized into primary amputation (PA), limb salvage (LS), and non-threatened limb trauma (NTLT) cohorts. The LS cohort was further divided into those with secondary amputation (LS-SA) and those without (LS-NA). The prevalence and incidence of 12 SHCs were analyzed across cohorts to test the hypotheses that (1) the prevalence of deleterious SHCs would differ among SMs with PA, LS, or NTLT, and (2) LS-SA would exhibit a greater prevalence of SHCs compared to LS-NA. **Results:** The prevalence of SHCs varied significantly across cohorts. Mental health disorders, nonspecific pain, and movement abnormalities were more prevalent in the PA cohort, while osteoarthritis, internal derangement of the knee, joint pain, and late-effect musculoskeletal injury were more prevalent in the LS cohort, specifically in the LS-SA subpopulation. The LS cohort had a higher prevalence of several SHCs than the NTLT cohort. Osteoarthritis incidence increased over time in all cohorts except NTLT, while unspecified back disorders decreased. Notable incidence differences were observed for late-effect musculoskeletal injury and other soft tissue disorders. **Conclusions:** This study characterizes SHCs associated with combat-related extremity trauma, emphasizing the need for tailored interventions and follow-up care based on specific injury management. Future research should explore underlying mechanisms and evaluate targeted interventions to minimize SHCs’ impact on patient outcomes.

## 1. Introduction

Musculoskeletal disorders are the leading cause of disability in both the United States (U.S.) civilian and military populations. In civilians, these conditions commonly arise after age 50 due to overuse or cumulative trauma. However, in the military, they often occur at much younger ages as a result of high-energy injuries. Advances in protective equipment and combat casualty care have increased survival rates from these severe injuries compared to past conflicts, resulting in a greater number of extremity injuries with unprecedented morbidity. Since 2001, over half of the combat injuries sustained by U.S. Service members (SMs) during overseas conflicts have affected their extremities [1,2]. Among these injuries, many threaten limb viability, necessitating a complex clinical management decision between amputation and limb salvage [3,4,5,6]. Several factors contribute to this decision-making process, including injury severity and available rehabilitation capabilities [7]. The extant literature comparing amputation and limb salvage following combat-related extremity injuries suggests that Service members undergoing limb salvage typically face higher rates of rehospitalization, surgical procedures, and complications [8,9,10,11,12,13,14,15,16]. These studies, however, have been limited by the inconsistent definition of limb salvage populations, hindering the accurate determination and utilization of data on limb salvage associated procedures and outcomes.

To address this issue, a data-driven approach to defining combat-related lower extremity limb salvage was developed, providing a validated, unbiased method for studying this population [17]. Moreover, by employing this data-driven approach, demographic characteristics and concomitant injuries of this limb salvage cohort were recently described [18]. While defining and describing Service members who underwent limb salvage after combat-related injuries represents important progress, a crucial next step is to understand the long-term consequences of these injuries and treatment decisions. Despite the dedicated efforts of highly integrated, interdisciplinary clinical teams providing complex care, individuals with severe extremity trauma remain at high risk of debilitating secondary health conditions. These conditions not only diminish their functional capacity and quality of life but also impose substantial costs on the Military Health System (MHS). These costs include direct medical expenses, medical retirements, and diminished Joint Force readiness [11].

As such, this study aims to comprehensively characterize the secondary health conditions (SHCs) associated with combat-related lower extremity limb salvage, given that the incidence and prevalence of SHCs within this cohort remain largely uninvestigated. We hypothesize significant differences in the prevalence of deleterious SHCs among U.S. Service members with combat-related lower extremity injuries who undergo primary amputation (PA), limb salvage (LS), or non-threatened limb trauma (NTLT). Furthermore, within the LS cohort, we anticipate a greater prevalence of SHCs in Service members who undergo secondary amputation (LS-SA) compared to those who do not (LS-NA). This research is expected to improve our ability to evaluate and treat these resultant health conditions, thereby reducing the burden on the Military Health System and enhancing the function and quality of life for Service members with these injuries.

## 2. Methods

### 2.1. Data Sources and Study Sample

This study utilized a previously described cohort [17] composed of SMs with combat-related injuries to the lower extremity who had acute injury encounters documented in the Expeditionary Medical Encounter Database [19] between 2004 and 2014.

### 2.2. Variables

The International Classification of Diseases, Ninth Revision (ICD-9) codes were used to identify SHCs present in the entire study population (Supplemental Table S1). ICD-9 codes that were not present in at least 5% of the population were excluded from the analysis. The ICD-9 codes which met the 5% threshold were categorized as follows: mental health disorders, hypertension, vertebral column disorders, back disorders (unspecified), unspecified pain, (i.e., pain not otherwise specified, Pain-NOS), osteoarthritis (upper and lower limbs), internal derangement of the knee, other derangements of the knee (upper and lower limbs), joint pain (upper and lower limbs), ligament/tendon disorders (both upper and lower limbs), disorders of muscle, ligament, and fascia, other soft tissue disorders (neuralgia and limb pain), acquired limb deformities, movement abnormalities, and late effects of musculoskeletal injuries.

### 2.3. Statistical Analysis

This study utilized data from the MHS Data Repository (MDR) to examine the frequency, type, and timing of SHCs in the first year following injury. The average number of SHC diagnoses, along with the standard deviation, was reported quarterly (0–3 months, 4–6 months, 7–9 months, and 10–12 months) across the three study groups. ANOVA was used to compare mean SHC diagnoses among study groups within each time frame, with Tukey post hoc comparisons performed upon detecting overall model significance. Prevalence was defined as the proportion of individuals within each population diagnosed with a specific SHC over the study duration, while incidence was calculated as the number of new diagnoses of particular SHCs within our populations of interest throughout the study period. Multivariate logistic regression analyses were conducted to identify SHCs associated with each cohort designation, with adjustments made for variables including age, polytrauma designation, mechanism of injury, and pre-existing diagnoses.

## 3. Results

### 3.1. Study Population

Within this cohort, 885 SMs underwent primary amputation (PA) within 15 days of injury, 2018 were identified as LS, and 1372 were identified as non-threatened limb trauma (NTLT). Among the LS cohort, 269 underwent secondary amputation (>15 days post injury; LS-SA) and 1749 had no amputation (LS-NA).

### 3.2. Prevalence of Secondary Health Conditions

The prevalence of diagnosis codes specific to SHCs within each of the injury cohorts was examined (Table 1). Out of the 16 SHCs investigated, 12 (75%) showed differences across the PA, LS, and NTLT cohorts (*p* ≤ 0.05). The most commonly diagnosed SHC in the PA cohort was other soft tissue disorders (75.8%), while joint pain was predominant in both the LS (71.4%) and NTLT cohorts (72.3%). Osteoarthritis of the upper limb consistently appeared as the least prevalent SHC across all cohorts, with no differences observed among them (*p* = 0.337).

Fisher’s exact tests highlighted a higher prevalence of mental health disorders (*p* = 0.003), Pain-NOS (*p* < 0.001), disorders of the muscle, ligament, and fascia (*p* < 0.001), other soft tissue disorders (*p* < 0.001), acquired limb deformities (*p* < 0.001), and movement abnormalities (*p* < 0.001) within the PA population relative to the LS population. Conversely, the LS population exhibited a higher prevalence of osteoarthritis (*p* < 0.001), internal derangement of the knee (*p* < 0.001), joint pain (*p* < 0.001), and late-effect musculoskeletal injury (*p* < 0.001) relative to the PA population.

Similarly, a higher prevalence of Pain-NOS (*p* < 0.001), osteoarthritis (*p* < 0.001), other joint derangement (*p* < 0.001), joint pain (*p* = 0.029), other soft tissue disorders (*p* < 0.001), and late-effect musculoskeletal injury (*p* < 0.001) was observed in the LS population relative to NTLT, with most of these differences stemming from diagnoses related to the lower limbs. None of the analyzed conditions showed increased prevalence in the NTLT population relative to the LS population.

Further subdivision of the LS population based on limb retention outcomes revealed differing prevalence rates among subpopulations for 7 of the 16 SHCs (Table 2). Specifically, mental health disorders (*p* = 0.001), Pain-NOS (*p* < 0.001), osteoarthritis (*p* = 0.023), disorders of muscle, ligaments, and fascia (*p* < 0.001), other soft tissue disorders (*p* < 0.001), movement abnormalities (*p* < 0.001), and late-effect musculoskeletal injury (*p* < 0.001) were found to be more prevalent in LS-SA than LS-NA. None of the analyzed SHC exhibited a greater prevalence in LS-NA relative to LS-SA.

### 3.3. Incidence of Secondary Health Conditions

**Figure 1:**
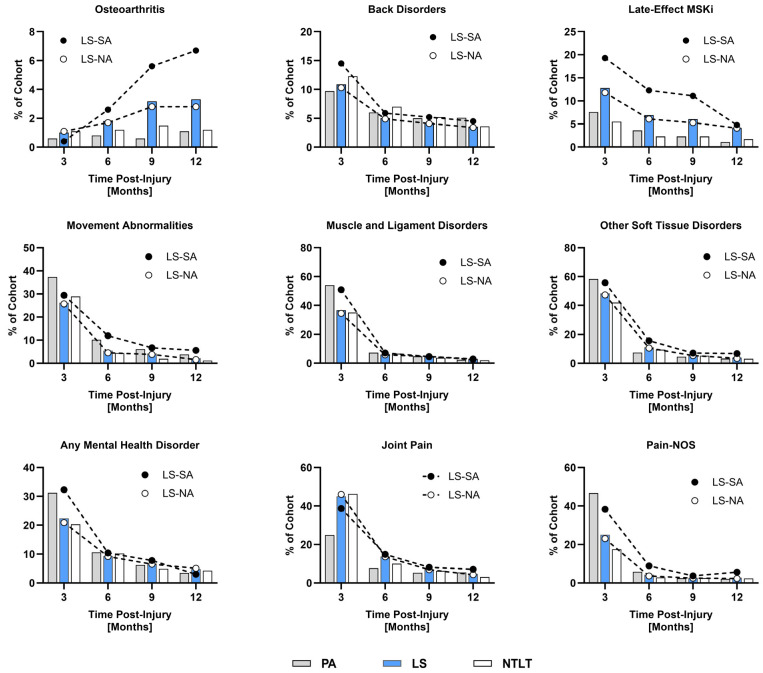
Incidence plots of various secondary health conditions observed in our extremity trauma cohorts.

The incidence rates for a subset of SHCs perceived to be most influenced by injury management strategies are depicted in Figure 1. Osteoarthritis incidence increased over time in every cohort except NTLT, with notable differences observed among the cohorts at 9 and 12 months. Conversely, rates of unspecified back disorders decreased over time with no discernible differences among the cohorts after 3 months. Although the incidence of late-effect musculoskeletal injury (MSKi) declined over time, rates of late-effect MSKi remained higher in the LS-SA population than the other cohorts for every time period studied. Overall, the incidence of movement abnormalities decreased over time in each cohort, with no differences in rates among the groups after 3 months. While there was a significant reduction in disorders of the muscle, ligament, and fascia across all cohorts, no disparities in rates among the groups were noted after 3 months.

The incidence of other soft tissue disorders also decreased over time, reaching just above 3% at 12 months in the PA (3.2%), LS-NA (3.4%), and NTLT (3.1%) cohorts; however, the incidence rate for the LS-SA cohort at 12 months was notably higher at 6.7%. Decreases in the incidence of joint pain were observed for each cohort over the course of a year, with differences noted at 3, 6, and 12 months. A substantial decrease in the incidence of pain not otherwise specified (NOS) was evident across all cohorts, with rates for PA, LS-NA, and NTLT nearly reaching 2% by 12 months, while the LS-SA cohort had a notably higher rate of 5.6%.

### 3.4. Odds Ratios

**Figure 2:**
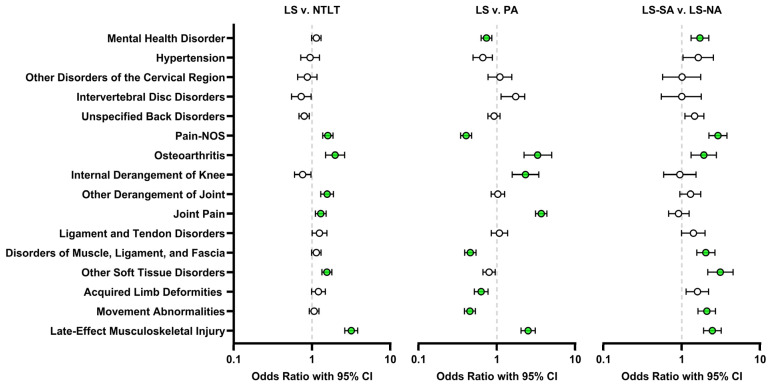
Forest plots of SHC odds ratios for the LS cohort relative to NTLT (**left**) and PA (**middle**) as well as for the LS-SA cohort relative to LS-NA (**right**). Statistically significant findings are highlighted in green (*p* < 0.05).

Odds ratios and 95% confidence intervals from the multivariate logistic regression models were used to evaluate the association of SHC with LS relative to the PA and NTLT comparison groups and with LS-SA relative to LS-NA (Figure 2). After adjusting for covariates, LS was found to have a higher likelihood of being diagnosed with pain-NOS, osteoarthritis, other joint derangement, joint pain, other soft tissue disorders, and late-effect musculoskeletal injury (*p* < 0.05 for all) relative to NTLT. LS did not exhibit a lower likelihood of diagnosis relative to NTLT for any of the SHCs studied. Outcomes relative to PA were more varied. Osteoarthritis (OR 3.32; *p* = 0.03), internal derangement of the knee, joint pain, and late-effect musculoskeletal injury were more likely to occur in LS relative to PA. In contrast, mental health disorders, pain-NOS, disorders of muscle, ligament, and fascia, acquired limb deformities, and movement abnormalities were lower in the LS cohort than in the PA cohort. Within the LS cohort, LS-SA were more likely to be diagnosed with mental health disorder; pain-NOS; osteoarthritis; disorders of muscle, ligament, and fascia; movement abnormalities; and late-effect musculoskeletal injury. LS-SA did not exhibit a lower likelihood of diagnosis relative to LS-NA for any of the SHCs studied.

## 4. Discussion

The analysis of SHCs among the PA, LS, and NTLT cohorts revealed several noteworthy findings that highlight the long-term health implications of different injury management strategies. Here, we discuss the prevalence, incidence, and odds ratios of SHCs across these cohorts, as well as the implications of these findings for clinical practice and future research directions.

As the research unfolded, it became abundantly clear that the landscape of SHCs following traumatic injury was complex and multifaceted. One striking revelation was the pervasive prevalence of mental health disorders across all groups studied. Regardless of whether individuals underwent PA and LS with or without limb retention, or experienced NTLT, mental health disorders loomed prominently, painting a poignant picture of the psychological toll of these injuries on individuals.

Another intriguing finding was the percentage of study participants diagnosed with vertebral or back conditions. Although there was a difference when comparing all groups, these diagnoses did not align with any specific injury management strategy when analyzed through odds ratios. This suggests that there may be complex interactions between injury types, treatment modalities, and subsequent health outcomes that require further investigation.

The prevalence of osteoarthritis in the LS subgroups, both with and without limb retention, was a concerning trend that emerged from our analysis. Limb salvage procedures, while aimed at preserving a patient’s natural limb and function, may set in motion a cascade of biomechanical and physiological changes that could be contributing to the elevated the risk of developing the osteoarthritis observed herein [20,21]. Joint resection and reconstruction often lead to abnormal joint loading and instability due to compromised soft tissues and altered limb alignment. While movement abnormalities were reduced in our LS population relative to our PA population, they were still more prevalent in the LS population than the NTLT population, and we can confidently assume that this would also be the case for a healthy population. Post-operative inflammation [22] and potential implant wear [23] are also known contributors to cartilage degradation, while reduced mobility and muscle weakness [24] can exacerbate joint stress, creating a complex interplay of factors that accelerate the degenerative process towards osteoarthritis in both the salvaged and the potentially unaffected contralateral limb.

Similarly, the persistence of late-effect musculoskeletal (MSK) injuries among both limb salvage subgroups throughout each observation period highlighted the need for continued support and interventions beyond the acute phase of injury. Despite advancements in medical technology and rehabilitation strategies, these individuals still face ongoing MSK issues. Interestingly, the comparison of movement abnormalities between the primary amputation and limb salvage with limb retention groups revealed a surprising parity. Despite the delay in amputation inherent in the LS-SA cohort, individuals in this group appeared to recover from movement abnormalities at a rate akin to those who underwent primary amputation. This unexpected symmetry underscored the adaptive capacity of the human body and the resilience inherent in the rehabilitation process. However, a concerning trend was observed in the limb salvage with limb retention group, with rates of pain increasing between the 9- and 12-month marks. This uptick in pain prevalence underscores the dynamic nature of post-injury recovery and emphasizes the importance of ongoing monitoring and intervention to address evolving health needs. Finally, the inverse relationship between primary amputation and pain in the joint offered a potential benefit of decisive surgical intervention. Individuals who underwent primary amputation were less likely to experience joint pain, suggesting that tailored treatment approaches may help mitigate the burden of SHCs and optimize long-term outcomes for those navigating traumatic injuries.

In sum, the exploration of SHCs following traumatic injury revealed a web of complexities, challenges, and occasional promising developments. The complexity of secondary health conditions (SHCs) following traumatic injury necessitates a nuanced approach in both policy and clinical practice. Policy implications include the potential need for resource allocation that acknowledges the varied SHC profiles between these populations. This may involve funding for specialized rehabilitation programs tailored to PA, LS, and NTLT cohorts, as well as long-term support services that address the chronic nature of many of these conditions. For instance, individuals with limb salvage might require early and intensive interventions for osteoarthritis, while those with amputations may benefit from specialized pain management and mental health support. Future research should focus on identifying optimal rehabilitation strategies for these disparate SHC profiles to inform evidence-based clinical guidelines.

## 5. Limitations

This study is reliant upon retrospective data, and as such the accuracy and reliability of the findings may be compromised by potential errors, inconsistencies, and missing data inherent in electronic medical records. The reliance on ICD-9 codes, the primary system during the data collection period, poses a limitation due to the potential for coding bias and misclassification. Coding bias may lead to inaccurate interpretations of disease incidence trends and could systematically under- or over-represent patient groups, thereby complicating comparative studies and obscuring genuine risk factors. While likely infrequent and uniformly distributed, misclassification errors within ICD-9 codes could also occur, potentially masking associations, weakening findings, skewing prevalence analyses, and resulting in misleading estimates of disease burden and false risk factors. Furthermore, the ICD-9 codes utilized in this study have been replaced by the more detailed ICD-10 system. Consequently, future research on the LS population employing the current definition will necessitate the conversion of the identified ICD-9 codes to their ICD-10 equivalents. This transition to ICD-10 demands a meticulous, expert-guided process because the mapping between the two systems is not one-to-one.

Aside from the limitations associated with the retrospective nature of this study, it should be noted that this study has limited generalizability as it focuses on a specific population of Service members with combat-related injuries to the lower extremity. Therefore, the findings may not be applicable to other populations, such as civilians or those with injuries to other body parts. It is also important to note that the study only examines SHCs occurring within the first year following injury. While this provides valuable insights into the early consequences of extremity trauma, it is crucial to recognize that some SHCs may develop beyond this time frame and the long-term consequences of trauma may not be fully captured in this study. Moreover, the study does not include data on functional outcomes, such as mobility, independence, or quality of life. These outcomes are essential for understanding the overall impact of trauma and the effectiveness of different injury management strategies. Additionally, the study does not provide detailed information on the treatments and rehabilitation strategies used for each cohort. This information would be valuable for understanding the factors contributing to the differences in SHCs among the groups and for informing future clinical practice. Finally, while the study adjusts for several confounding factors, such as age, polytrauma designation, mechanism of injury, and pre-existing diagnoses, other factors may also influence the development of SHCs. These may include socioeconomic status, education level, and access to healthcare services, which are not accounted for in this study.

## 6. Conclusions

These findings have significant implications for clinical practice and emphasize the importance of tailored interventions and follow-up care based on the specific injury management strategy employed. Clinicians should be cognizant of the heightened risk of certain secondary health conditions associated with primary amputation or limb salvage and incorporate comprehensive assessment and management strategies into patient care plans. Future research should focus on uncovering the underlying mechanisms behind the development of SHCs following different injury management strategies and evaluating the effectiveness of targeted interventions in minimizing their impact on patient outcomes.

## Figures and Tables

**Table 1:** Prevalence of secondary health conditions across injury groups.

Secondary Health Effect Diagnoses	Primary Amputation N = 885		Limb Salvage N = 2018		Non-Threatened Limb Trauma N = 1372		*p*-Values		
	*f*	%	*f*	%	*f*	%	Χ^2^ Test	Fisher’s Exact Test	
								LS v. PA	LS v. NTLT
Mental Health Disorder	479	54.1	938	46.5	594	43.3	<0.001	0.003	0.598
*Adjustment disorder*	301	34.0	537	26.6	332	24.2	<0.001	0.001	0.716
*Anxiety states*	159	18.0	306	15.2	192	14.0	0.226	0.436	0.978
*Depression*	131	14.8	294	14.6	166	12.1	0.337	0.971	0.466
*Post-traumatic stress disorder*	199	22.5	539	26.7	359	26.2	0.261	0.163	0.992
Hypertension	86	9.7	134	6.6	96	7.0	0.107	0.065	0.992
Other disorders of the cervical region	48	5.4	119	5.9	92	6.7	0.421	0.971	0.978
Intervertebral disc disorders	27	3.0	105	5.2	96	7.0	0.003	0.140	0.403
Unspecified back disorders	242	27.3	519	25.7	416	30.3	0.111	0.892	0.061
Pain-NOS	505	57.1	706	35.0	346	25.2	<0.001	<0.001	<0.001
Osteoarthritis	27	3.0	191	9.5	69	5.0	<0.001	<0.001	<0.001
*Upper limb*	3	0.3	21	1.0	10	0.7	0.337	0.436	0.978
*Lower limb*	19	2.1	149	7.4	51	3.7	<0.001	<0.001	<0.001
Internal derangement of knee	31	3.5	157	7.8	137	10.0	<0.001	<0.001	0.398
Other derangement of joint	170	19.2	397	19.7	186	13.6	<0.001	0.971	<0.001
*Upper limb*	95	10.7	92	4.6	68	5.0	<0.001	<0.001	0.992
*Lower limb*	80	9.0	294	14.6	121	8.8	<0.001	<0.001	<0.001
Joint pain	423	47.8	1557	77.2	992	72.3	<0.001	<0.001	0.029
*Upper limb*	183	20.7	368	18.2	299	21.8	0.224	0.546	0.197
*Lower limb*	293	33.1	1440	71.4	891	64.9	<0.001	<0.001	0.002
Ligament/tendon disorders	108	12.2	263	13.0	147	10.7	0.337	0.971	0.490
*Upper limb*	53	6.0	83	4.1	61	4.4	0.337	0.302	0.992
*Lower limb*	43	4.9	161	8.0	72	5.2	0.007	0.033	0.045
Disorders of muscle, ligament, and fascia	614	69.4	1028	50.9	655	47.7	<0.001	<0.001	0.598
Other soft tissue disorders	671	75.8	1441	71.4	846	61.7	<0.001	0.161	<0.001
*Neuralgia*	115	13.0	188	9.3	123	9.0	0.034	0.053	0.992
*Pain in limb*	606	68.5	1310	64.9	756	55.1	<0.001	0.436	<0.001
Acquired limb deformities	188	21.2	293	14.5	169	12.3	<0.001	<0.001	0.598
Movement abnormalities	508	57.4	765	37.9	500	36.4	<0.001	<0.001	0.978
Late-effect musculoskeletal injury	129	14.6	605	30.0	163	11.9	<0.001	<0.001	<0.001

**Notes**: *f*—frequency; %—percentage. Highlighting indicates significant findings (*p* < 0.05).

**Table 2:** Prevalence of secondary health conditions within the limb salvage group.

Secondary Health Effect Diagnoses	Limb Salvage Secondary Amputation LS-SA N = 269		Limb Salvage No Amputation N = 1749		Fisher’s Exact *p*-Values
	*f*	%	*f*	%	
Mental Health Disorder	156	58.0	782	44.7	0.001
*Adjustment disorder*	88	32.7	449	25.7	0.247
*Anxiety states*	55	20.4	251	14.3	0.205
*Depression*	59	21.9	235	13.4	0.012
*Post-traumatic stress disorder*	76	28.2	463	26.5	0.999
Hypertension	26	9.7	108	6.2	0.515
Other disorders of the cervical region	16	5.9	103	5.9	1.000
Intervertebral disc disorders	14	5.2	91	5.2	1.000
Back disorders, unspecified	87	32.3	432	24.7	0.146
Pain-NOS	154	57.2	552	31.6	<0.001
Osteoarthritis	41	15.2	150	8.6	0.023
*Upper limb*	2	0.7	19	1.1	1.000
*Lower limb*	33	12.3	116	6.6	0.049
Internal derangement of knee	20	7.4	137	7.8	1.000
Other derangement of joint	63	23.4	334	19.1	0.745
*Upper limb*	15	5.6	77	4.4	0.997
*Lower limb*	44	16.4	250	14.3	0.997
Pain in joint	204	75.8	1353	77.4	0.999
*Upper limb*	45	16.7	323	18.5	0.999
*Lower limb*	191	71.0	1249	71.4	1.000
Ligament/tendon disorders	45	16.7	218	12.5	0.604
*Upper limb*	14	5.2	69	3.9	0.991
*Lower limb*	24	8.9	137	7.8	0.999
Disorders of muscle, ligament, and fascia	177	65.8	851	48.7	<0.001
Other soft tissue disorders	235	87.4	1206	68.9	<0.001
*Neuralgia*	38	14.1	150	8.6	0.122
*Pain in limb*	217	80.7	1093	62.5	<0.001
Acquired limb deformities	54	20.1	239	13.7	0.124
Movement abnormalities	144	53.5	621	35.5	<0.001
Late-effect musculoskeletal injury	129	48.0	476	27.2	<0.001

**Notes**: *f*—frequency; %—percentage. Highlighting indicates significant findings (*p* < 0.05).

## Data Availability

All data supporting the findings of this study are available within the paper and its Supplementary Information.

## Supplementary Materials

The following supporting information can be downloaded at: https://www.mdpi.com/article/10.3390/jcm14103472/s1, Table S1: Prevalence of secondary health effects among total extremity trauma population.

## Copyright Statement

This manuscript is a U.S. Government work that was prepared as part of the official duties of U.S. Government employees. Title 17, U.S.C., Section 105 provides that copyright protection is not available for any work of the U.S. Government.

## References

[1] Cross J.D., Ficke J.R., Hsu J.R., Masini B.D., Wenke J.C. (2011). Battlefield orthopaedic injuries cause the majority of long-term disabilities. J. Am. Acad. Orthop. Surg..

[2] Fischer H. (2015). A guide to US military casualty statistics: Operation freedom’s sentinel, operation inherent resolve, operation new dawn, operation Iraqi freedom, and operation enduring freedom. Congr. Res. Serv..

[3] Roessler M.S., Wisner D.H., Holcroft J.W. (1991). The mangled extremity. When to amputate?. Arch. Surg..

[4] Prasarn M.L., Helfet D.L., Kloen P. (2012). Management of the mangled extremity. Strateg. Trauma Limb. Reconstr..

[5] Lange R.H. (1989). Limb reconstruction versus amputation decision making in massive lower extremity trauma. Clin. Orthop. Relat. Res..

[6] Hansen S.T. (1989). Overview of the severely traumatized lower limb. Reconstruction versus amputation. Clin. Orthop. Relat. Res..

[7] MacKenzie E.J., Bosse M.J., Kellam J.F., Burgess A.R., Webb L.X., Swiontkowski M.F., Sanders R., Jones A.L., McAndrew M.P., Patterson B. (2002). Factors influencing the decision to amputate or reconstruct after high-energy lower extremity trauma. J. Trauma.

[8] Melcer T., Sechriest V.F., Walker J., Galarneau M. (2013). A comparison of health outcomes for combat amputee and limb salvage patients injured in Iraq and Afghanistan wars. J. Trauma Acute Care Surg..

[9] Melcer T., Walker J., Bhatnagar V., Richard E., Sechriest V.F., Galarneau M. (2017). A Comparison of Four-Year Health Outcomes following Combat Amputation and Limb Salvage. PLoS ONE.

[10] Farrokhi S., Mazzone B., Eskridge S., Shannon K., Hill O.T. (2018). Incidence of Overuse Musculoskeletal Injuries in Military Service Members with Traumatic Lower Limb Amputation. Arch. Phys. Med. Rehabil..

[11] Butowicz C.M., Dearth C.L., Hendershot B.D. (2017). Impact of Traumatic Lower Extremity Injuries Beyond Acute Care: Movement-Based Considerations for Resultant Longer Term Secondary Health Conditions. Adv. Wound Care.

[12] Bosse M.J., Ficke J.R., Andersen R.C. (2012). Extremity war injuries: Current management and research priorities. J. Am. Acad. Orthop. Surg..

[13] Dagum A.B., Best A.K., Schemitsch E.H., Mahoney J.L., Mahomed M.N., Blight K.R. (1999). Salvage after severe lower-extremity trauma: Are the outcomes worth the means?. Plast. Reconstr. Surg..

[14] Reiber G.E., McFarland L.V., Hubbard S., Maynard C., Blough D.K., Gambel J.M., Smith D.G. (2010). Servicemembers and veterans with major traumatic limb loss from Vietnam war and OIF/OEF conflicts: Survey methods, participants, and summary findings. J. Rehabil. Res. Dev..

[15] Harris A.M., Althausen P.L., Kellam J., Bosse M.J., Castillo R. (2009). Complications following limb-threatening lower extremity trauma. J. Orthop. Trauma.

[16] Busse J.W., Jacobs C.L., Swiontkowski M.F., Bosse M.J., Bhandari M. (2007). Complex limb salvage or early amputation for severe lower-limb injury: A meta-analysis of observational studies. J. Orthop. Trauma.

[17] Goldman S.M., Eskridge S.L., Franco S.R., Souza J.M., Tintle S.M., Dowd T.C., Alderete J., Potter B.K., Dearth C.L. (2023). A Data-Driven Method to Discriminate Limb Salvage from Other Combat-Related Extremity Trauma. J. Clin. Med..

[18] Goldman S.M., Eskridge S.L., Franco S.R., Dearth C.L. (2023). Demographics and Comorbidities of United States Service Members with Combat-Related Lower Extremity Limb Salvage. J. Clin. Med..

[19] Galarneau M.R., Hancock W.C., Konoske P., Melcer T., Vickers R.R., Walker G.J., Zouris J.M. (2006). The Navy-Marine Corps Combat Trauma Registry. Mil. Med..

[20] Ogilvie C.M., Crawford E.A., Hosalkar H.S., King J.J., Lackman R.D. (2009). Long-term results for limb salvage with osteoarticular allograft reconstruction. Clin. Orthop. Relat. Res..

[21] Heijink A., Gomoll A.H., Madry H., Drobnič M., Filardo G., Espregueira-Mendes J., Van Dijk C.N. (2012). Biomechanical considerations in the pathogenesis of osteoarthritis of the knee. Knee Surg. Sports Traumatol. Arthrosc..

[22] Sokolove J., Lepus C.M. (2013). Role of inflammation in the pathogenesis of osteoarthritis: Latest findings and interpretations. Ther. Adv. Musculoskelet. Dis..

[23] Goodman S.B., Gallo J., Gibon E., Takagi M. (2020). Diagnosis and management of implant debris-associated inflammation. Expert Rev. Med. Devices.

[24] Nelson C.M., Marchese V., Rock K., Henshaw R.M., Addison O. (2020). Alterations in Muscle Architecture: A Review of the Relevance to Individuals After Limb Salvage Surgery for Bone Sarcoma. Front. Pediatr..

