# Supplementary material for: Characterization of Secondary Health Conditions Among United States Service Members with Combat-Related Lower Extremity Limb Salvage
Source: J Clin Med. 2025 May 15;14(10):3472. doi: 10.3390/jcm14103472 (PMC12112558; doi:10.3390/jcm14103472)
Supplement: Supplementary file 1 [file jcm-14-03472-s001.zip › jcm-3590318-supplementary.pdf]

Table S1. Prevalence of secondary health effects among total extremity trauma population

Secondary Health Effect	ICD-9 code	n (% of total population n=4275)
Depression*	296 (.2, 3), 300.4, 311	592 (13.8)
Anxiety states*	300.0	657 (15.4)
Other pain disorders related to psychological factors	307.89	75 (1.7)
Adjustment disorder*	309 (except 309.81)	1170 (27.4)
PTSD*	309.81	1097 (25.7)
Pain, NOS	338 (.0-.2, .4)	1557 (36.4)
Cauda equina syndrome	344.6	45 (1.0)
Lumbosacral root lesions, NOS	353.4	8 (0.2)
Phantom limb	353.6	786 (18.4)
Carpel tunnel	354.0	121 (2.8)
Lesion of sciatic nerve	355.0	161 (3.8)
Hypertension	401	316 (7.4)
Osteoarthritis	715	287 (6.7)
Osteoarthritis- UE	715 (.11-.14, .21-.24, .31-.34, .91-94)	34 (0.8)
Osteoarthritis- LE	715 (.15-.17, .25-.27, .35-.37, .95-97)	219 (5.1)
Traumatic arthopathy	716.1	197 (4.6)
Traumatic arthropathy- UE	716 (.11-.14)	28 (0.6)
Traumatic arthropathy-LE	716 (.15-.17)	132 (3.1)
Internal derangement of knee	717	325 (7.6)
Other derangement of joint	718 (.0, .2 - .4, .5, .8)	753 (17.6)
Other derangement of joint- UE	718 (.01-.04, .21-.24, .31-.34, .41-.44, .51-.54, .81-.84)	255 (6.0)
Other derangement of joint- LE	718 (.05-.07, .25-.27, .35-.37, .45-.47, .55-.57, .85-.87)	495 (11.6)
Other and unspecified disorders of joint	719	3608 (84.4)
Effusion/hemarthrosis	719 (.0, .1)	224 (5.2)
Effusion/hemarthrosis-UE	719 (.01-.04, .11-.14)	19 (0.4)
Effusion/hemarthrosis-LE	719 (.05-.07, .15-.17)	201 (4.7)
Pain in joint	719.4	2972 (69.5)
Pain in joint-UE	719 (.41-.44)	850 (19.9)
Pain in joint-LE	719 (.45-.47)	2624 (61.4)

Ankylosing spondylitis/other inflammatory spondylopathies	720	43 (1.0)
Spondylosis and allied disorders	721	85 (2.0)
Intervertebral disc disorders	722	228 (5.3)
Other disorders of cervical region	723 (.1-.3, .9)	259 (6.1)
Other and unspecified disorders of back	724	1177 (27.5)
Other backpain	724 (.1-.5)	1021 (23.9)
Disorders Ligament/tendon to bone	726	518 (12.1)
Disorders Ligament/tendon to bone- UE	726 (.0-.4)	197 (4.6)
Disorders Ligament/tendon to bone-LE	726 (.5 - .7)	276 (6.5)
Other disorders of synovium tendon and bursa	727 (.0, .2-.9)	78 (1.8)
Other disorders of synovium tendon and bursa-UE	727 (.03-.05, .62-.64)	77 (1.8)
Other disorders of synovium tendon and bursa-LE	727 (.06, .65-.68)	78 (1.8)
Disorders of muscle ligament and fascia	728	2297 (53.7)
Other disorders of soft tissues	729 (.2, .4-.9)	2958 (69.2)
Neuralgia	729.2	426 (10.0)
Pain in limb	729.5	2672 (62.5)
Major Osseos defects	731.3	46 (1.1)
osteochondropathy, unspecified	732.8	1 (0.02)
Osteoporosis	733.0	74 (1.7)
Pathological fracture	733.1	106 (2.5)
Stress fractures	733.93-98	44 (1.0)
Flat foot	734	58 (1.4)
Acquired limb deformities	736 (.3, .7-.9)	650 (15.2)
Scoliosis & lordosis	737(.2, .3, .43, .8)	21 (0.5)
Other acquired deformity; spine	738 (.4-.6)	36 (0.8)
non-allopathic lesions, lumbar/sacral	739 (.3-.4)	129 (3.0)
Movement abnormalities	781 (.2 - .4)	1773 (41.5)
Late effect musculoskeletal injury	905 (.3-.9)	897 (21.0)
